# Hospital transmission of borderline oxacillin-resistant *Staphylococcus aureus* evaluated by whole-genome sequencing

**DOI:** 10.1099/jmm.0.001384

**Published:** 2021-07-16

**Authors:** Maria M. Konstantinovski, Karin Ellen Veldkamp, Adriana P. M. Lavrijsen, Thijs Bosch, Margriet E. M. Kraakman, Sam Nooij, Eric C. J. Claas, Jairo Gooskens

**Affiliations:** ^1^​ Medical Microbiology Department, Leiden University Medical Center, Leiden, Netherlands; ^2^​ Dermatology Department, Leiden University Medical Center, Leiden, Netherlands; ^3^​ Infectious Diseases Research, Diagnostics and Laboratory Surveillance, National Institute for Public Health and the Environment, de Bilt, Netherlands

**Keywords:** antimicrobial resistance, BORSA, cgMLST, infection control, nosocomial transmission, WGS

## Abstract

**Introduction:**

*Staphylococcus aureus* is a major cause of hospital infections worldwide. Awareness towards methicillin-resistant *S. aureus* (MRSA) infections is high but attention towards borderline oxacillin-resistant *S. aureus* (BORSA) is limited, possibly due to an underestimated clinical relevance, presumption of low incidence and diagnostic limitations.

**Gap statement:**

BORSA surveillance has not been routinely implemented, and thus consensus with regard to a definition and infection control measures is lacking.

**Aim:**

Our goals were to investigate the occurrence, molecular characteristics and clinical manifestations of BORSA infections in the hospital setting.

**Methodology:**

Following an increased incidence in 2016, BORSA cases in 2014/2016 (in our institution) were more specifically evaluated. Medical records were reviewed to investigate epidemiological links, clinical characteristics and outcomes. Resistance and virulence markers were assessed by whole genome sequencing (WGS). Conventional methods: amplified fragment length polymorphism (AFLP) ; multilocus sequence typing (MLST) and multiple locus variable-number tandem repeat analysis (MLVA) were compared with core genome MLST (cgMLST) and whole-genome single nucleotide polymorphism (wgSNP) analysis to confirm genetic clusters.

**Results:**

From 2009 to 2013, BORSA comprised 0.1 % of all clinical *S. aureus* strains. In 2016, the incidence was six-fold higher in comparison to the baseline. Whole-genome SNP and cgMLST confirmed two BORSA clusters among patients with dermatological conditions. Patients with BORSA presented with skin infections, and one case developed a severe invasive infection with a fatal outcome. Infection control measures successfully prevented further transmission in both clusters. WGS findings showed that BORSA strains carried multiple resistance and virulence genes with increased pathogenic potential.

**Conclusion:**

WGS and cgMLST effectively characterized and confirmed BORSA clusters among at-risk patients with clinical manifestations ranging from mild skin infections to life-threatening bacteraemia. Clinical awareness and active monitoring are therefore warranted for the timely implementation of infection control measures to prevent BORSA transmission in high-risk patients.

## Introduction

*Staphylococcus aureus* is a common bacterial pathogen that causes a wide range of community-acquired and healthcare-associated infections. β-Lactam antibiotics are the treatment of choice as most *S. aureus* are susceptible to penicillinase-resistant penicillins, including oxacillin and methicillin [1]. The increasing occurrence and transmission of methicillin-resistant *S. aureus* (MRSA) in hospital settings are of concern due their association with more difficult-to-treat infections and increased mortality [2]. Effective infection prevention and control measures are important to prevent ongoing transmission to vulnerable patients and healthcare workers (HCW) [3, 4].

MRSA carry modified penicillin-binding protein (PBP) 2a or 2c with a low affinity to β-lactam antibiotics encoded by *mecA* or *mecC* genes [5]. Thus, antibiotic options are severely limited with vancomycin being the first-choice regimen for severe infections requiring parental treatment [6, 7]. Unfortunately, vancomycin has a slower bactericidal rate against staphylococci and is inferior when compared to β-lactam treatment of severe *S. aureus* infections such as bacteraemia and endocarditis [8–12].

Another type of oxacillin-resistant *S. aureus* is the borderline oxacillin-resistant *S. aureus* (BORSA) phenotype that contains alternative resistance mechanisms. These strains include β-lactamase-hyperproducing *S. aureus* and *S. aureus* with point mutations in other PBP genes [13–18]. There are no strict laboratory diagnostic criteria to define these resistance phenotypes. Other diagnostic limitations include oxacillin breakpoint guidelines differences (EUCAST, >2 mg l^−1^; CLSI, ≥4 mg l^−1^) and the use of cefoxitin, which fails to detect oxacillin resistance due to production of β-lactamase. The clinical relevance of these resistance mechanisms is of debate, and with a presumption of limited clinical relevance. A lack of a definition and urgency have led to the fact that BORSA infection control measures and surveillance have not been routinely implemented [19]. However, β-lactam antibiotics are not proven to be effective for the treatment of severe BORSA infections, and reports of treatment failures exist [20, 21].

In this outbreak analysis, we evaluated the occurrence and clinical manifestations of BORSA infections in the hospital setting, after an increase in incidence in 2016. We performed whole-genome sequencing (WGS) of all isolates for an assessment of the genetic relatedness of the presumed outbreak isolates using two different methods, core genome multilocus sequence typing (cgMLST) and a whole genome single nucleotide polymorphism (wgSNP) analysis.

The primary objective of this study was to elucidate the role and clinical relevance of nosocomial BORSA transmission using molecular typing tools.

## Methods

### Setting and data collection

Leiden University Medical Centre (LUMC) is a tertiary medical centre with an average of 25 000 admissions per year.

The Infection Control department initiated an outbreak investigation in May 2016 following consecutive BORSA infection cases at the Dermatology outpatient clinic. This outbreak analysis includes eight clinical BORSA isolates obtained from patients presenting at LUMC from January 2014 to December 2016 with a BORSA infection. Medical records were reviewed to obtain relevant clinical data concerning the BORSA infection, the infection date, site, treatment and outcome, and to investigate common exposures and an epidemiological link between the cases. All Dermatology HCW and other contacts of BORSA-positive patients were screened for BORSA carriage to investigate transmission.

The study describes routine outbreak investigation procedures initiated by the Infection Control department, and permission from the Ethics board was obtained to process and present this information.

### BORSA definition

BORSA isolates were defined as *S. aureus* with an oxacillin MIC ≥2 mg l^−1^ as measured with an *E*-test (bioMérieux) and without the presence of *mecA* and *mecC* genes.

### Routine diagnostics and antimicrobial susceptibility testing

Clinical cultures were ordered by the attending physician, and processed in the laboratory as per routine. In the case of a clinically relevant culture with *S. aureus,* susceptibility testing was performed using a Vitek2 Gram-Positive Susceptibility card (AST-P633; bioMérieux), which included susceptibility results for oxacillin, clindamycin, vancomycin, rifampicin and fusidic acid, and a cefoxitin screening.

If the Vitek2 oxacillin MIC value was ≥4 mg l^−1^, an *E*-test was performed using an inoculum of a McFarland 0.5–1.0 suspension in saline and incubated for 24 h on Mueller Hinton agar with 2 % NaCl, in ambient air at 35 °C. To assess the presence of *MecA/C* genes, a PCR (BDmax StaphSR PCR Assay; BD Diagnostics) was performed on all strains with an oxacillin *E*-test value of ≥2 mg l^−1^.

### Contact screening cultures

All dermatology HCW were consequently screened for BORSA carriage when an outbreak was suspected. HCW provided a swab from the throat and nose, and, if applicable, skin defects. Patient contacts were also screened for BORSA carriage and provided a throat, nose and perineum swab [22]. Swabs were incubated in a Brain Heart Infusion broth with 2.5 % NaCl and colistin. The broth was plated on ChromID agar plates (bioMérieux), and on selected colonies, a *MecA/C* PCR (BDmax StaphSR PCR Assay; BD Diagnostics) was performed after 18–24 h of incubation.

Susceptibility testing was performed as described previously.

### Conventional molecular typing methods

Molecular typing was used in the outbreak investigation to assess the relatedness of the BORSA strains.

AFLP (amplification fragment length polymorphism) analysis was performed in our institution using a previously described protocol [23]. AFLP patterns were analysed using BioNumerics software, version 7.1 (Applied Maths NV), and the similarity between normalized AFLP patterns (range 60–600 bp) was calculated with the Pearson product-moment correlation coefficient and UPGMA (unweighted pair group method with arithmetic mean) algorithms. Strains with more than 90 % similarity were considered to be closely related.

Both multiple loci variable-number tandem repeat analysis (MLVA) and MLST was performed in the national reference centre for MRSA, The National Institute for Public Health and the environment [24, 25]. MLST is performed using DNA nucleotide sequences of seven housekeeping genes (*arcC, aroE, glpF, gmk, pta, tpi, yqi*) and analysed using http://saureus.mlst.net/

### WGS methods

We performed WGS of all BORSA strains as part of the outbreak investigation. The genetic relatedness of these strains was analysed using two different methods, cgMLST and a whole genome SNP (wgSNP) analysis. A BORSA control strain with a similar conventional typing result was added before calculating a minimun spanning tree. WGS was also used to investigate the presence of virulence and antimicrobial resistance genes. Mutations in the *gdpP*, *PBP4* and *BlaZ* genes were assessed. The WGS typing results were compared with conventional AFLP and MLVA typing tools.

Bacterial strains were cultured on Columbia blood agar at 37 °C. A McFarland 3.0 suspension was prepared from a fresh isolate for DNA extraction using the QIAsymphony DSP Virus/Pathogen Midi kit (Qiagen) with an input of 800 µl. The sequence libraries were prepared using a NEBNext Ultra II DNA Library Prep Kit (New England Biolabs) for a 150 bp paired-end sequencing run on the Illumina NextSeq500. Sample preparation and WGS data analysis were performed in our institution, whereas the sequencing run was performed at GenomeScan laboratories.

For cgMLST analysis, raw sequence data were transferred to a FASTA file and analysed using SeqSphere+ software version 6.0.2 (Ridom) [26]. The number of core-genome targets for *S. aureus* is 1861 with a Cluster Alert of 24 differences or more [27].

For SNP analysis, we used the Basty pipeline (version 0.9.0) from BIOPET [28] to produce multisample vcf files. Using this multisample vcf file, the SNP distances between strains were calculated using VCFtools (version 0.1.16; with parameter ‘--relatedness2’) [29]. The distances between strains were visualized with R software version 3.6.1 [30] using the pheatmap package [31] based on the manhattan clustering distance. More details regarding this pipeline can be found in the supplementary material.

To detect mutations in the *gdpP, PBP4* and *BlaZ* genes, we aligned the genes to each strain with the software program Geneious (version 10.2.6) using sequences from blast.

VirulenceFinder and ResFinder web-based tools [32, 33] were used to find the virulence- and antimicrobial resistance genes (see Table 1). The Resistance Gene Identifier (RGI), which relies on the Comprehensive Antibiotic Resistance Database (CARD), was used to find antimicrobial resistance genes for all samples [34].

**Table 1.** Molecular typing and susceptibility results of outbreak strains

BORSA	MLVA typing	MLST	MLST profile	cgMLST	Oxacillin MIC range*	Phenotypic susceptibility	VirulenceFinder	ResFinder and CARD
Patient 1	MT5355 – MC045 Cluster 2	ST45-like CC45	10, 14, 8, 6, 10, 3, ?	CT3242	3-4	Cli S, Cotrim S, Fus R, Rif S, Vanco S, Fox S	*aur, sak, scn, hlgA, hlgB, hlgC, hlb, seg, sei, sel, sem, sen, seo, seu, sec3*	*blaZ, norA, mepA,* *mepR, tet38,* *sav1866, arlS, arlR*
Patient 2	MT0272 – MC08	ST8 CC8	3, 3, 1, 1, 4, 4, 3	CT3248	2	Cli R, Cotrim S, Fuc S, Rif S, Vanco S, Fox S	*aur, splA, splB, splE, sak, scn, hlgA, hlgB, hlgC, hlb, lukD, lukE*	*norA, mepA, mepR* *tet38, sav1866, arlS*
Patient 3	MT5355 – MC045 Cluster 2	ST45-like CC45	10, 14, 8, 6, 10, 3, ?	CT3242	3-8	Cli S, Cotrim S, Fuc R, Rif S, Vanco S, Fox S	*aur, sak, scn, hlgA, hlgB, hlgC, hlb,* *seg, sei, sel, sem, sen, seo, seu, sec3*	*blaZ, norA, mepA* *mepR, tet38, sav1866* *arlS, arlR*
Patient 4	MT0272 – MC08 Cluster 1	ST8-like CC8	3, 3, 1, 1, ?, 4, 3	CT3243	2-4	Cli R, Cotrim S, Fuc S, Rif S, Vanco S, Fox S	*aur, splA, splB, splE, hlgA, hlgB, hlgC, hlb, lukD, lukE*	*blaZ, norA, ermC* *mepA, mepR, tet38* *sav1866, arlS*
Patient 5	MT0272 – MC08 Cluster 1	ST8-like CC8	3, 3, 1, 1, ?, 4, 3	CT3243	2-3	Cli R, Cotrim S, Fuc S, Rif S, Vanco S, Fox S	*aur, splA, splB, splE, hlgA, hlgB, hlgC, hlb, lukD, lukE*	*blaZ, norA, ermC* *mepA, mepR, tet38* *sav1866, arlS*
Patient 6	MT0272 – MC08 Cluster 1	ST8-like CC8	3, 3, 1, 1, ?, 4, 3	CT3243	2	Cli R, Cotrim S, Fuc S, Rif S, Vanco S, Fox S	*aur, splA, splB, splE, hlgA, hlgB, hlgC, hlb, lukD, lukE*	*blaZ, norA, ermC* *mepA, mepR, tet38* *sav1866, arlS*
Patient 7	MT0272 – MC08 Cluster 1	ST8-like CC8	3, 3, 1, 1, ?, 4, 3	CT3243	6	Cli R, Cotrim S, Fuc S, Rif S, Vanco S, Fox S	*aur, splA, splB, splE, hlgA, hlgB, hlgC, hlb, lukD, lukE*	*blaZ, norA, ermC* *mepA, mepR, tet38* *sav1866, arlS*
									Patient 8	n/a	ST34 CC30		CT6016	4	Cli S Cotrim S, Fuc S, Rif S, Vanco S, Fox R	n/a	n/a

*Range of MIC (mg l^−1^) as measured with an Etest of all *S. aureus* strains from the patient that showed Vitek2 >4 mg l^−1^ for oxacillin.

## Results

### Epidemiology

In 2016, an increased number of BORSA isolates were reported over a period of several months, leading to suspicion that there may have been nosocomial transmission occurring. In this period, five consecutive BORSA patients presented among 750 *S. aureus*-positive patients, an incidence of 0.66. In 2009–2015 preceding the start of the outbreak investigation, the incidence of BORSA was only 0.1 % with eight BORSA patients among 8345 *S*. *aureus*-positive patients identified. This increase in BORSA incidence was statistically significant, with a *P*-value of <0.001 as calculated with the Pearson chi-squared test.

### Outbreak investigation

The Infection Control Unit initiated an outbreak investigation in 2016 following the epidemiological increase of BORSA in early 2016. During this investigation, patients with a BORSA-positive culture in 2016 were retrospectively reviewed. This investigation revealed that the outbreak may have started as early as 2014, as epidemiological links with the Dermatology department among BORSA cases from 2014 to 2016 were identified. A total of eight BORSA patients from this period were identified of whom seven had contact with the Dermatology department (Table 2). Six out of eight patients visited the Dermatology Outpatient Clinic between January and May 2016, and one patient visited the Dermatology Outpatient Clinic only once in 2015. Two out of eight patients received light therapy involving twice weekly visits to the clinic, and several times their visits were scheduled on coinciding days. Other epidemiological links included two patients who were admitted to the hospital and were seen by the same consulting Dermatologist, on the same dates but on different wards. Two patients were admitted to the same Internal medicine ward on overlapping days, but did not share a room.

**Table 2.** Clinical overview of BORSA cases involved in 2014–2016 outbreak analysis

Patient	Year	Medical history	BORSA infection	Antibiotic pretreatment
P1: F, 52y	2014	Keratosis follicularis	Cellulitis of both ears with alternating BORSA-positive cultures and chronic BORSA carrier	Flucloxacillin oral course
P2: M, 76y	2015	Recurrent squamous cell carcinoma	Infection of wound after excision of skin carcinoma	Cotrimoxazol oral course. Fusidic acid topical course
P3: F, 46y	2016	Acute undifferentiated leukaemia, 6 months after SCT, GvHD skin	Cellulitis, secondary infecion of GvHD of the skin, small abscess of the axilla	Cotrimoxazol oral course
P4: M, 27y	2016	Eczema, aortic prosthetic valve due to congenital heart disease	Recurrent MSSA endocarditis with involvement of prosthetic material. Patient was initially treated with high-dose flucloxacillin and during the fourth episode of recurrence a BORSA was identified and treatment switched to vancomycin after 5 days. Fatal outcome	Three high-dose flucloxacillin i.v. courses of 6–8 weeks followed by oral clindamycin
P5; F, 58y	2016	Psoriasis, SLE, diabetic foot	Infected ulcers on the foot, cellulitis	Flucloxacillin and clindamycin oral course
P6: M, 22y	2016	Eczema	Infected eczema	No pre-treatment
P7: M, 79y	2016	Late-onset eczema	Ecthyma form of impetigo located on the hand	No pre-treatment
P8: M, 23Y	2015	Kidney transplantation	*S. aureus* bacteraemia and possible endocarditis originating from an infected venous line, complete recovery	Unkown

Year, most recent year with positive cultures available; antibiotic pretreatment, antibiotics received in the year prior to BORSA infection; F, female; M, male; y, years; SCT, stem cell transplantation; GvHD, graft versus host disease; SLE, systemic lupus erythematosis.

Patient 8 seemed epidemiologically unrelated, had no contact with the dermatology department and carried a strain with a distinct susceptibility pattern; for these reasons, this patient was excluded from further outbreak analysis. Individual phenotypic susceptibility results of the outbreak cluster strains are given in Table 1.

The strains were isolated from a wide range of specimens, and a clinical overview of patients is depicted in Table 2. All patients manifested (recurrent) skin infections, and one case (patient 4) developed a severe invasive infection with a fatal outcome. Several patients received β-lactam antibiotics in the year prior to the first positive BORSA culture.

A common source was suspected, and several infection control measures were initiated at the Dermatology Outpatient Clinic. Contact isolation precautions for positive patients were applied. Hand hygiene compliance and the importance of individualized use of creams and lotions was emphasized. A total of 47 dermatology HCW were screened, but their cultures revealed no BORSA carriage. No new cases were identified during a 6-month follow-up period after the outbreak investigation.

### Molecular typing andwhole-genome sequence analysis

Conventional typing methods identified two different clusters by using AFLP, MLVA, and MLST (Table 2). The BORSA strains from patients 2, 4, 5, 6 and 7 had similar AFLP patterns, MLVA profiles MT0272 and MLSTST8(-like) results and were assigned to Cluster 1. BORSA strains isolated with a 14-month time interval from patients 1 and 3 had similar AFLP patterns, MLVA MT 5355 profiles, and MLST ST45-like results, and were assigned to Cluster 2.

Further analysis based on whole-genome sequence result showed successful extraction >97% of cgMLST targets in all strains.

Cluster 2 cgMLST results confirmed that isolates from patients 1 and 3 were related with a genetic distance of only 5 alleles and formed one genetic cluster that belonged to CT3242. SNP analysis showed a slightly larger genetic distance of 113 SNP. The exact moment of transmission between these patients is unknown, however both patients visited the Dermatology outpatient clinic on consecutive days for a routine check up.

**Fig. 1.**
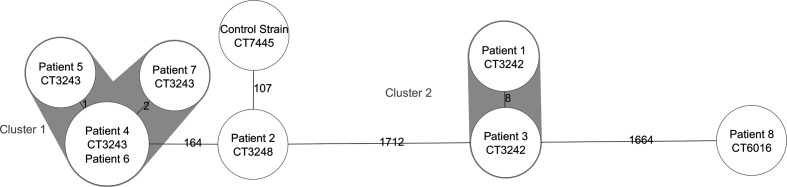
Minimum spanning tree, calculated with Seqsphere+ using the outbreak strains. The complex type is noted within the nodes. Allelic distances are noted on the connection lines (not to scale).

The cgMLST results for Cluster 1 isolates from patient 2, 4, 5, 6 and 7 could not confirm one genetic cluster. Isolates 4, 5, 6 and 7 belonged to CT3243 with a genetic distance ranging from 0 to 2 allelic differences and patient 2 belonged to another complex type, CT3248. (Fig. 1). The genetic distance between the complexes was 163 alleles, and the presence of additional resistance genes in CT3243 (ermC) and absence of virulence genes *sak*, *scn* and resistance gene *BlaZ* suggested a high genetic heterogeneity.

**Fig. 2.**
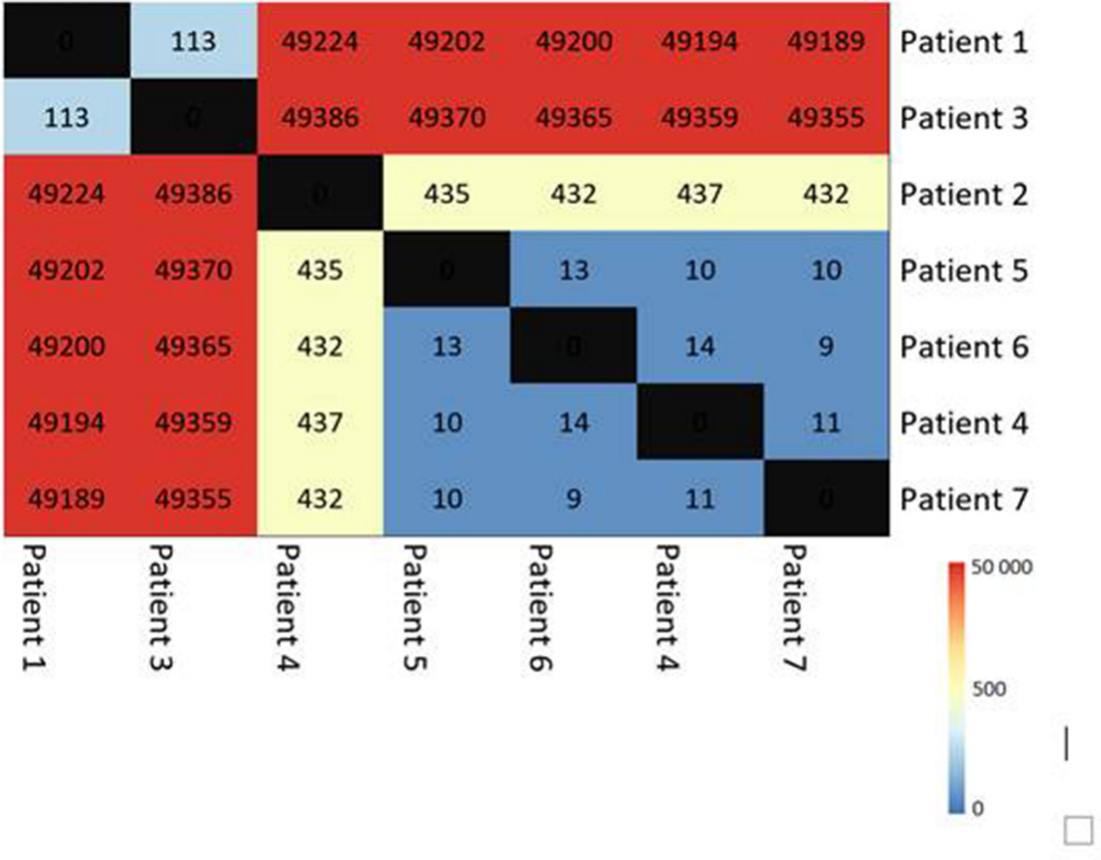
Overview of SNP differences between the BORSA strains, visualized with pheatmap. Colors indicate the SNP distance between strains.

Whole-genome SNP analysis showed that the differences ranged from 5 to15 SNP between the strains from patients 4, 5, 6 and 7 as can be seen in Fig. 2.

Patient 8 belonged to CT6016 and was highly unrelated with a genetic distance of 1664 alleles or more.

In total, cgMLST showed 4 distinct genetic lineages, including two clusters (CT3242 and CT3243) and two singletons (CT3248 and CT6016; Fig. 1).

Whole-genome sequence analysis using CARD and VirulenceFinder showed that BORSA isolates from 2014 to 2016 carried multiple virulence factors and resistance genes, as summarized in Table 2. Cluster strains harbored β-lactamase, quinolone, and macrolide resistance genes. The wide range of virulence genes encoded for aureolysins, serine proteases, staphylokinases, leukocidins, b-and g-hemolysins, superantigens, complement inhibitors, and enterotoxins.

Analysis of mutations in the *gdpP*, *PBP4* and *blaZ* genes reveiled that these genes were identical within each cluster and carried several mutations.

Cluster 1 *gdpP* gene had an insertion at N263I. The cluster 1 *PBP4* gene had 6 mutations: F12C, A25T, R101T, S189T, A398E and A409T. The Cluster 1 *blaZ* gene had a E112A mutation, and belonged to type A.

Cluster 2 *gdpP* showed mutations N263I, I152V en I456V .The cluster 2 *PBP4* gene had 6 mutations: F12C, R101T, S189T, Y208F, V381F and R430I. The *blaZ* belonged to type C.

## Discussion

In this study, WGS analysis confirmed hospital transmission events during an increase from the baseline incidence, and our findings highlight that clinically relevant outbreaks can occur among high-risk patients. Skin conditions are a known risk factor for the acquisition and transmission of *S. aureus,* including MRSA. This is especially the case for atopic dermatitis [35–38].

Previous BORSA outbreaks have been suggested by others, using conventional molecular techniques, including PFGE, but these methods have a relatively low discriminatory power and cluster confirmation is therefore limited. Our results provided evidence that a strain that seemed related to a cluster by AFLP and MLVA was unrelated using WGS data with 162 allelic cgMLST and 435 wgSNP differences. cgMLST established itself as a reliable and user-friendly tool that can be used for prospective surveillance and comparison between laboratories and differentiate with a higher resolution between relatedness due to transmission events compared to conventional molecular techniques [27, 39–41]. In our study, cgMLST confirmed four different genetic lineages and two clusters. wgSNP analysis confirmed the relatedness of the strains and may have the potential to provide even more in-depth data as it takes into account all genetic differences between strains. A cgMLST scheme counts only one allelic change when multiple nucleotide changes within the same gene are observed. Thus, SNP analysis may yield a higher number of differences between strains. This will probably explain why the number of SNP differences between the strains is slightly higher than the number of allelic changes. This is shown in patients 4 and 6, who are typed as identical by cgMLST but have 14 SNP differences. As we do not known the exact sequalea of transmission it is unknown whether the SNP differences are related to the number of events that were needed for the strains to spread from the source. The genetic distance within our clusters ranged from one to five allelic differences, which is within the eight allelic differences criterion defining recent *S. aureus* transmission as proposed by others [40, 42].

BORSA outbreaks have previously been reported among dermatology patients [43, 44]. All BORSA patients in our outbreak analysis had a history of dermatological conditions. Infection control investigations at the Dermatology department and outpatient clinic indicated possible epidemiological links. Culturing of HCW revealed no source of transmission and environmental samples could not be obtained due to the time that elapsed. It remains unclear what caused the increase in cases and how transmission could have occurred. Potentially shared creams or lotions could be a point source. However, a diversity of potential epidemiological links was identified, as some patients were admitted to the same ward, were seen by the same physician on different wards or had light therapy on the same day. It is plausible that transmission routes varied among patients, and multiple modes of transmission are of importance when it comes to preventative measures for this specific patient group. Fortunately, early reinforcement of infection control measures halted ongoing nosocomial spread.

Recent surveillance studies suggest a BORSA incidence in hospital settings of approximately 1–5 % [13, 45–47]. We describe a lower incidence, but this may be due to underreporting in our study. Diagnostic limitations may include the routine use of automated susceptibility testing methods with no active BORSA screening thresholds, the lack of addition of NaCl in routine cultures to improve the synthesis of β-lactamases and application of cefoxitin susceptibility testing for oxacillin resistance screening.

No exact criteria exist to define a BORSA phenotype, and oxacillin MIC thresholds ranging from 1 to 8 mg l^−1^ have been suggested. However, most authors classify *S. aureus* with an MIC of at least 2–4 mg l^−1^ as borderline resistant for oxacillin [13]. EUCAST clinical breakpoints suggest breakpoints above 2 mg l^−1^ for oxacillin as suspect for either BORSA or MRSA. This is in line with our findings, where most isolates had an oxacillin MIC of 2–4 mg l^−1^. Future studies may focus on optimizing BORSA detection using automated susceptibility testing by defining thresholds for oxacillin that could trigger more accurate MIC testing in selected isolates. It is interesting to note that in one of the patients, one strain belonging to the same genotypic cluster exhibited MICs ranging from 2 to 6 mg l^−1^ and showed documented treatment failure. These findings suggest a threshold of maximum 4 mg l^−1^ in automated testing and adopting an MIC of 2 mg l^−1^ or higher for oxacillin as measured by an *E*-test to define BORSA.

In the literature, a β-lactam at high dose is suggested as a therapeutic option for the treatment of uncomplicated BORSA infections [20, 48–52]. Our findings indicate a risk of therapeutic failure using high-dose β-lactam for the treatment of severe BORSA infections, including those causing endocarditis. The initial treatment of a case of BORSA endocarditis consisted of 12 g/24 h i.v. flucloxacillin. It was switched to vancomycin after 5 days when susceptibility results showed an oxacillin MIC of 4 mg l^−1^, a susceptible cefoxitin screen and absence of *MecA/C* which excluded MRSA. High-dose β-lactam treatment failure was reported in a previous case of BORSA endocarditis [21]. Both cases demonstrated *in vivo* development of oxacillin resistance after initial high-dose β-lactam treatment and subsequent recurrence. Caution should be exercised when prescribing β-lactam treatment for BORSA infections.

Molecular characterization showed that all BORSA isolates carried a wide range of resistance and virulence markers. Interestingly, the cluster strains had several mutations in the *PBP4* and *gdpP* genes. Some of those mutations (Y208F, V381F and R430I) have been associated with decreased oxacillin susceptibility by Argudin *et al.* [53]. Cluster 1 strains carried a mutation in *blaZ* E112A that very recently has been associated with increased oxacillin resistance [54]. BORSA isolates may be characterized into β-lactamase-hyperproducing *S. aureus* (BORSA), modified *S. aureus* with point mutations in PBP genes expressing a reduced affinity for β-lactams (MODSA) and other mechanisms [13, 45, 55]. Evaluation of more genetic markers is an object for future studies [53]. Several questions remain unanswered concerning the emergence and genetic diversity of BORSA in nosocomial and community settings. Further understanding of the molecular epidemiology and resistance mechanisms of BORSA is of clinical relevance [56] and can be accomplished by implementing systematic surveillance programmes and by establishing a local reference database. Further research should focus on identifying the underlying molecular mechanisms for oxacillin resistance in BORSA.

In conclusion, our findings highlight that clinically relevant BORSA outbreaks can occur in a low-incidence hospital setting and that WGS-based analysis can be used for accurate outbreak confirmation. This should be confirmed in studies with higher numbers, as our outbreak included only a low study number to investigate.

Implementing cgMLST and reference databases in infection control practice will aid in defining transmission pathways and improving prevention and control measures to avoid transmission to HCW and vulnerable patients.

## Supplementary Data

Supplementary material 1
