# Supplementary material for: Hospital transmission of borderline oxacillin-resistant Staphylococcus aureus evaluated by whole-genome sequencing
Source: J Med Microbiol. 2021 Jul 16;70(7):001384. doi: 10.1099/jmm.0.001384 (PMC8493421; doi:10.1099/jmm.0.001384)
Supplement: Supplementary material 1 [file jmm-70-1384-s001.pdf]

Hospital transmission of Borderline oxacillin-resistant *Staphylococcus aureus* (BORSA) evaluated by whole-genome sequencing (WGS)

M.M. Konstantinovski[1], K.E. Veldkamp[1], A.P.M Lavrijsen[1], T.Bosch[2], M.E.M. Kraakman[1], S.Nooij[1], E.C.J Claas[1], J. Gooskens[1]

Affiliations:

1 Leiden University Medical Center, Leiden, Netherlands

2 National Institute for Public Health and the Environment, de Bilt, Netherlands

Corresponding author: Konstantinovski,

Keywords

BORSA, infection control, WGS, cgMLST, nosocomial transmission, antimicrobial resistance

Supplemental material

Description of SNP analysis using Basty pipeline

A concise description of the Basty pipeline with regards to the included steps.

First, the pipeline does a quality control of raw reads with fastqc (version 0.10.1; http://www.bioinformatics.babraham.ac.uk/projects/fastqc/), then removes adapters from raw reads with cutadapt (version 1.10; Martin, online at https://doi.org/10.14806/ej.17.1.200) and quality trimming with sickle (version 1.33, default parameters; *Joshi NA, Fass JN. 2011, Sickle: A sliding-window, adaptive, quality-based trimming tool for FastQ files*, Available at https://github.com/najoshi/sickle.) then aligns remaining, high quality reads with bowtie2 (version 2.3.4.1; Langmead & Salzberg, 2012 (https://doi.org/10.1038/nmeth.1923)) to the reference genome of *S. aureus* subsp. aureus COL (GenBank accession ID NC_002951.2). From the alignment a vcf file is created using samtools mpileup (version 1.1; parameters '-u -v') and bcftools call (version 1.1-134; parameters '-v -c -f GQ'). (*Heng Li, A statistical framework for SNP calling, mutation discovery, association mapping and population genetical parameter estimation from sequencing data Bioinformatics. 2011 Nov 1; 27(21): 2987–2993.).*
